# String Method with Swarms-of-Trajectories, Mean Drifts, Lag Time, and Committor

**DOI:** 10.1021/acs.jpca.1c04110

**Published:** 2021-08-18

**Authors:** Benoît Roux

**Affiliations:** † Department of Biochemistry and Molecular Biology, The University of Chicago, Chicago, Illinois 60637, United States; ‡ Department of Chemistry, The University of Chicago, 5735 S. Ellis Avenue, Chicago, Illinois 60637, United States

## Abstract

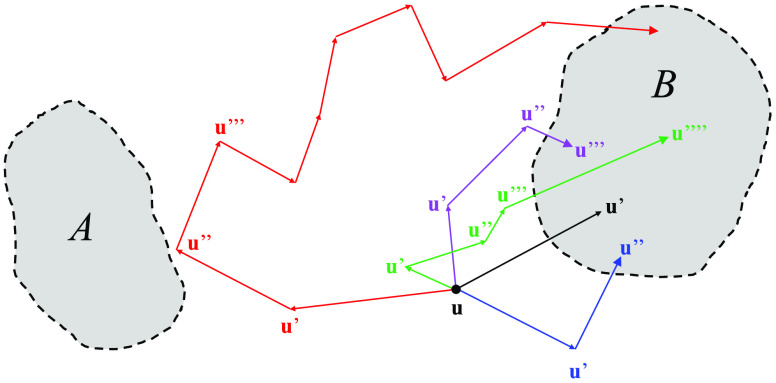

The kinetics of a dynamical system comprising two metastable states is formulated in terms of a finite-time propagator in phase space (position and velocity) adapted to the underdamped Langevin equation. Dimensionality reduction to a subspace of collective variables yields familiar expressions for the propagator, committor, and steady-state flux. A quadratic expression for the steady-state flux between the two metastable states can serve as a robust variational principle to determine an optimal approximate committor expressed in terms of a set of collective variables. The theoretical formulation is exploited to clarify the foundation of the string method with swarms-of-trajectories, which relies on the mean drift of short trajectories to determine the optimal transition pathway. It is argued that the conditions for Markovity within a subspace of collective variables may not be satisfied with an arbitrary short time-step and that proper kinetic behaviors appear only when considering the effective propagator for longer lag times. The effective propagator with finite lag time is amenable to an eigenvalue-eigenvector spectral analysis, as elaborated previously in the context of position-based Markov models. The time-correlation functions calculated by swarms-of-trajectories along the string pathway constitutes a natural extension of these developments. The present formulation provides a powerful theoretical framework to characterize the optimal pathway between two metastable states of a system.

## Introduction

A central problem in computational biophysics is the characterization of the long-time kinetic behavior of molecular systems. Many of the key concepts can be formulated by considering a prototypical system comprising two dominant metastable states A and B. In the context of a multistate Markov model, the steady-state flux from A to B can be expressed as the net sum of productive transitions across a dividing surface between the two end states. Further analysis shows that the steady-state probability of the states under such nonequilibrium conditions can be expressed as the product of the equilibrium probability of the states times the probability that a trajectory initiated at the same position will be reactive and first reach the state B before ever reaching the state A.^1^ This “committor” probability, which can be determined on the basis of the backward dynamical propagation, then becomes a critical ingredient in efforts to formulate a theoretical framework seeking to treat such problems.^1,2^

This analysis leads to the observation that the principal lines of a reactive probability current between the states A and B are largely determined by the equilibrium probability times the local gradiant of the committor.^2^ This observation provides a critical insight in the formulation of the string method,^3−5^ which seeks to determine the dominant “reaction tube” that contains most of the probability current between A and B. Mathematically, the line of maximum probability flux is a curve in the phase space, and a calculation of the full committor is practically infeasible. To reduce the complexity of the problem, Maragliano et al.^6^ assumed that the committor probability depends predominantly on a subset of collective variables (CVs), **z** ≡ {*z*_1_, *z*_2_, ..., *z*_*N*_}. These considerations provide the background that led to the development of the string method with CVs on the potential of mean force (PMF) surface *W*(**z**).^6^ The string method represents the curvilinear minimum free energy pathway (MFEP) linking the states A and B as a curve in the space of the collective variables. Inspired by pioneering work from Pratt^7^ and Elber and Karplus,^8^ this curve (the string) is constructed iteratively as a chain of *M* copies of the system (“images”). In its original formulation, the information needed for each image is the mean force and the value of a metric tensor, both of which can be expressed in terms of conditional averages. This algorithm has been employed in a variety of applications.^9−17^

The problem of optimizing the path in a multidimensional space, nonetheless, remains arduous and computationally expensive. To circumvent these difficulties, a number of variations of the original mean force algorithm of Maragliano et al.^6^ have been proposed, including the finite temperature string method,^18^ the string method with swarms-of-trajectories,^19^ and multiscale preconditioning.^20^ The idea of the string method with swarms-of-trajectories^19^ (also called the “drift” method^21^) is to rely on the outcome from a large number of short unbiased trajectories launched from the positions of the images along the curve to determine the optimal pathway. Because it relies on a large number of independent trajectories, the algorithm scales extremely well on large supercomputers,^22^ and this becomes especially effective with applications to hybrid quantum mechanical-molecular mechanical (QM/MM) simulations due to the poor scaling displayed by ab initio codes.^23^ The method has been used to characterize a conformational transition in very large macromolecular systems, including the activation of c-Src tyrosine kinase^24,25^ cholesterol flip-flop in lipid membranes,^26^ the movement of the voltage-sensor of K^+^ channels,^27^ the alternating-access mechanism in the sarcoplasmic reticulum calcium pump (SERCA),^28^ and the chemomechanical coupling in V-type ATPases.^29^

It has been shown that the original mean force method of Maragliano et al.^6^ and the swarms algorithm^19^ are essentially equivalent in the limit of very short trajectories.^30^ However, the significance of the swarms algorithm for longer trajectories remains unclear. Intuitive arguments relying on a physical picture of overdamped diffusional dynamics suggest that the mean drifts from the swarms-of-trajectories may have the ability to more correctly capture the effective behavior of the system supporting the construction of meaningful transition pathways.^19,21^ But the lack of a rigorous theoretical treatment has prevented the analysis to go any further.

The goal of the present effort is to return to this unresolved issue and clarify the fundamental underpinning of the string method with swarms-of-trajectories. After a brief summary of the developments leading to the original mean force string method,^6^ we begin by formulating the problem of the kinetics of a dynamical system comprising two metastable states A and B in terms of a finite-time propagator in phase space (position and velocity) adapted to the underdamped Langevin equation. This development is inspired by previous work based on propagators for position-based Markov models.^31−34^ From this formulation, an effective propagator in the space of the CVs is defined, and its properties are clarified. It is shown that a formulation of the propagator with a finite lag time helps to clarify the conditions for Markovity within the subspace of the CVs. On the basis of this analysis, the significance of thea string method with swarms-of-trajectories with finite lag time is clarified.

## Analysis

### String Method in Collective Variables

For the sake of clarity and completeness, we briefly recall the formal developments leading to the original mean force string method.^6^ We first consider a system evolving according to the underdamped Langevin equation
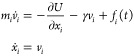
1with ⟨*f*_*i*_⟩ = 0 and ⟨*f*_*i*_(*t*)*f*_*j*_(*t*′)⟩ = 2*γk*_B_*Tδ*_*ij*_δ(*t* – *t*′). The generic friction γ is assumed to be very small and is introduced primarily to act as a weak thermostat. The equilibrium distribution is ρ_eq_(**x**,**v**) = *e*^–*H*(**x**,**v**)/*k*_B_*T*^/∫ d**x** d**v***e*^–*H*(**x**,**v**)/*k*_B_*T*^, where *H*(**x**,**v**) = *U*(**x**) + ∑_*i* = 1_^*N*^ 1/2*m*_*i*_*v*_*i*_^2^ is the Hamiltonian.

It is assumed that the system comprises two metastable states A and B (defined by the user). The forward committor *q*_+_^B^(**x**,**v**) is the probability that a path starting at (**x**,**v**) ultimately reaches the state B before ever reaching the state A. The committor *q*_+_^B^(**x**,**v**) is defined as , with the constraints *q*_+_^B^(**x**, **v**) = 0 when (**x**, **v**) ∈ A, and *q*_+_^B^(**x**, **v**) = 1 when (**x**, **v**) ∈ B, where  is the backward Kolmogorov propagator.
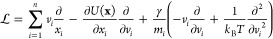
2The forward committor *q*_+_^B^(**x**, **v**) can be also obtained through a variational formulation by seeking to minimizes *I*(2,6)
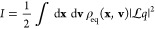
3over all trial functions *q*(**x**, **v**) satisfying the constraints for the state A and B.^6^

The string method of Maragliano et al.^6^ represents the pathway linking A and B as a “chain of state”, that is, a collection of *M* images located at the positions {**z**^1^, ..., **z**^*M*^} in a subspace of collective variables (CVs). Here, **z** represents a vector-valued function, **z̃**(**x**) = (*z̃*_1_(**x**), ...,*z̃*_*N*_(**x**)), that maps every configuration **x** of the system on a set of values **z̃**(**x**). The central asantz to derive the string method in CVs is to assume a trial committor *q̅* that depends only on **z**.^6^ Inserting *q̅*(**z**) in eq 3, *I* is reduced as
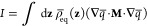
4with **∇***q̅* = (∂*q̅*/∂*z*_1_, ∂*q̅*/∂*z*_2_, ...). The reduced equilibrium probability ρ̅_eq_(**z**) is defined in terms of *W*(**z**), the PMF associated with these variables
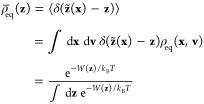
5and the components of **M**(**z**) are given by the conditional average
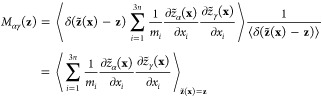
6Having performed a dimensionality reduction leading to eq 4, one then seeks to minimize *I* over all functions *q̅* subject to the constraint *q̅* = 0 when **z** ∈ A, and *q̅* = 1 when **z** ∈ B. The Euler–Lagrange equation for the effective committor *q̅*(**z**) is
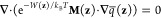
7subject to the constraint *q̅*(**z**) = 0 when **z** ∈ A and *q̅*(**z**) = 1 when **z** ∈ B. The MFEP corresponds to the curve (or line) linking the states A and B defined such that its tangent follows the direction of the vector **v**.
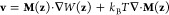
8The curve can be generated, for example, by starting from a saddle point in the space **z** and following the direction **v** in small increment or, equivalently, by building up a curve such that the projection of **v** in the direction perpendicular to its tangent vanishes, that is, [**v**]^⊥^ = 0. The term **∇**·**M**(**z**) is often negligible, and in this case, the MFEP is a curve tangent to the vector **M**(**z**)·∇*W*(**z**) in the space of the collective variables that connects the two states A and B. These results summarize the mean force string method in collective variables of Maragliano et al.^6^ Conceptually, the developments leading to this theory represent one formal “route” to achieve a dimensionality reduction to the subspace of the CVs by inserting *q̅*(**z**) in eq 3.

For the sake of argument, let us assume that, by virtue of the particular system of interest, it is a physically valid approximation to treat **z** as a set of slow variables. In other words, the CVs genuinely undergo an overdamped dynamics on the free energy surface *W*(**z**) with a diffusion coefficient *D*_α_ γ(**z**) according to the Smoluchowski equation. This statement of fact notwithstanding, both eq 7 for the effective committor *q̅*(**z**) and eq 8 defining the tangent to the curvilinear path underlying the mean force string method remain unchanged. On the one hand, the mean force string method based on eqs 5, (6), and (8) is effectively “blind” to the nature of the microscopic dynamics of the CVs. On the other hand, since we know with confidence that the CVs are indeed slow variables, the committor *q̅*(**z**) can be determined from the backward Kolmogorov equation (see eq 2.5 in ref (35))
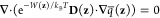
9subject to the constraint *q̅*(**z**) = 0 when **z** ∈ A and *q̅*(**z**) = 1 when **z** ∈ B. Accordingly, the optimal path should be determined by
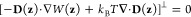
10This treatment treating the CVs as slow variables represents a different formal “route” to achieve a dimensionality reduction to the subspace of the CVs. Clearly, a comparison of eqs 7 and (9) shows that the two routes do not yield the same result.

When the dimensionality is reduced by an insertion of the asantz *q̅*(**z**) in eq 3, the optimal path is determined from the mean force string method eq 8. In this case, the path is affected by the PMF and the matrix **M**. In contrast, when the CVs are slow and the problem is first reduced to an overdamped diffusional dynamics, the optimal path is determined from eq 10. In this case, the path is affected by the potential *W* and the diffusion matrix **D**. While the matrices **D** and **M** occupy a similar place in the equation defining the committor *q̅*(**z**), **D** is associated with dynamical dissipative effects, whereas **M** is a mass-weighted geometric factor reporting the change in the curvilinear collective variables elicited by a corresponding change in the Cartesian coordinates. For instance, the matrix **M** is simply constant and diagonal if the CVs are Cartesian variables. Specifically, the matrix **M** does not incorporate dynamical dissipative effects, which would be needed to characterize the CVs as slow variables.

Fundamentally, the discrepancy stems from the transition from underdamped inertial dynamics to overdamped diffusional dynamics. Somehow, the analysis that leads to eq 7 does not lead to eq 9 when the CVs are genuinely slow variables. Traditionally, the behavior of slow degrees of freedom is revealed through an analysis of an effective dynamics based on projection operators.^36−38^ The expectation is that the slow dissipative dynamics of the CVs should be reflected in the time evolution of the system. However, to display the dynamical evolution of the CVs, the propagation of the system over a finite time is required, and this information is not available with the operator  in the variational principle expressed by eq 3. An alternative route is needed to perform this analysis. In the following, we will reconstruct the steps leading to the committor in a subspace of CVs in terms of a finite-time propagator for this purpose.

### Microscopic Propagator

The underdamped Langevin dynamics eq 1 is prescribed by the Green’s function propagator. The theoretical formulation seeking to build up the kinetic behavior from a finite-time propagator in phase space (positions and velocities) is inspired by previous developments of Markov model position-propagators.^31−34^ Here, this general picture is expanded to underdamped Langevin dynamics. The probability density of the system at time *t* is expressed as ρ(**x**,**v**; *t*), where **x** and **v** represent the set of coordinates *x*_*i*_ and velocities *v*_*i*_, respectively. Using **u** ≡ (**x**,**v**) as a shorthand to represent the point in phase space, the forward propagation step (**u** →**u**′) for the probability density from the time *t* to the time *t* + Δ*t* is
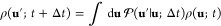
11The elementary propagator for a null time step, , is the identity δ(**u**′ – **u**), and the dynamical propagation, which we may formally represent as , obeys the Chapman-Kolmogorov equation for arbitrary times. The implication is that, while Δ*t* is a microscopic time step (e.g., 1 fs), the propagator may be repeatedly applied an arbitrary number of times as , with
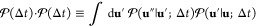
12The backward propagation step is . Probability is conserved by both the forward propagator  and backward propagator , implying that the unity function is a left eigenvector of these operators with an eigenvalue equal to 1. The total energy accumulated over the propagation correspond to the heat transferred from the bath to the system from the Langevin propagation, *Q* = *H*(**u**′) – *H*(**u**). Because *Q* is the heat exchanged with the reservoir during the forward propagation step, it follows that −*Q* is the heat exchanged with the reservoir for a propagation along the reverse step. Noting that *H*(**x**,**v**) = *H*(**x**,–**v**), and ρ_eq_(**u**) = ρ_eq_(**x**,–**v**), the forward–backward microscopic detailed balance relation
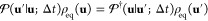
13is satisfied. The momentum reversal requirement for microscopic reversibility is discussed in section 2.2.3 of ref (39). It follows that the canonical equilibrium distribution is invariant by propagation
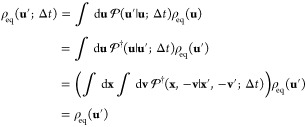
14Thus, , showing that **ρ**_eq_ is a right-eigenvector with an eigenvalue equal to 1.

### Committor Probabilities for Two Metastable States

**Figure 1:**
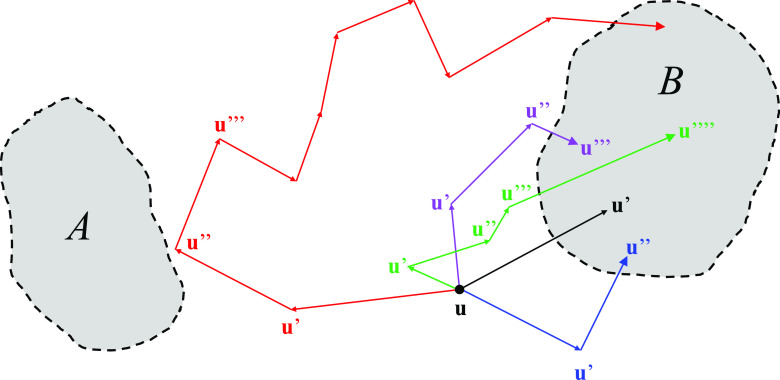
Schematic illustration of the forward committor *q*_+_^B^(**u**) represented as the sum over all possible paths starting at **u** that ultimately reach the state B before ever reaching the state A. All paths start at **u** and end in B without entering the state A. The black path reaches the B region in one step (**u** → **u**′), the blue path in two steps (**u** → **u**′ → **u**″), the purple path in three steps (**u** → **u**′ → **u**″ → **u**‴), and the green path in four steps (**u** → **u**′ → **u**″ → **u**‴ → **u**‴′). The restriction that the intermediate states resulting from all these steps are ∉ A,B applies to all paths. For example, the longer red path does not enter the boundary region of state A before reaching the state B in the final destination.

Assuming two metastable states A and B, the forward committor *q*_+_^B^(**u**) is the sum of the probability over all paths starting at **u** that ultimately reach the state B before ever reaching the state A. The probability of each of these paths is expressed as a product of discrete propagation steps , under the restriction that the intermediate states resulting from all these steps are ∉ A, B. The sum over paths is illustrated schematically in Figure 1. It follows that *q*_+_^B^(**u**) is written explicitly as
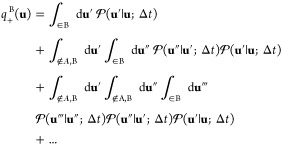
15
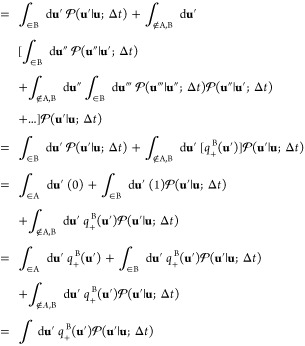
16with the constraints of *q*_+_^B^(**u**) = 0 if **u** ∈ A and *q*_+_^B^(**u**) = 1 if **u** ∈ B. By construction, 0 ≤ *q*_+_^B^(**u**) ≤ 1. This expression may be related to eq 2 by recognizing that the operator  is defined in the limit of an infinitesimal time step.
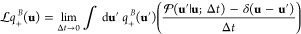
17

A forward committor *q*_+_^A^(**u**) can also be defined, satisfying the constraints *q*_+_^A^(**u**) = 1 if **u** ∈ A and *q*_+_^A^(**u**) = 0 if **u** ∈ B and the identity *q*_+_^A^(**u**) + *q*_+_^B^(**u**) = 1. In a similar fashion, a backward committor probability *q*_–_^A^(**u**) can be defined from the backward propagation as
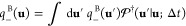
18with the constraints of *q*_–_^B^ = 0 if **u** ∈ A and *q*_–_^B^ = 1 if **u** ∈ B. A backward committor *q*_–_^A^(**u**) can be defined, satisfying the identity *q*_–_^A^(**u**) + *q*_–_^B^(**u**) = 1. Furthermore, it can be verified that *q*_–_^A^(**u**) = *q*_+_^A^(**x**,–**v**) and *q*_–_^B^(**u**) = *q*_+_^B^(**x**,–**v**).

While the equations for the committor probabilities involve only the elementary propagator  for the short time Δ*t*, the fundamental validity of these equations is predicated upon the necessity to satisfy the Markovity of the dynamics as expressed by the Chapman-Kolmogorov equation .

### Steady-State Flux between Two States

This section largely follows the development previously laid out by Berezhkovskii, Hummer, and Szabo^40^ for continuous-time discrete-state Markov models. A number of established relations are briefly recapped for the sake of completeness. To elicit a net nonequilibrium flux from a metastable state A to metastable state B, we construct steady-state conditions as^40^
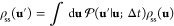
19with ρ_ss_(**u**) = ρ_eq_(**u**) if **u** ∈ A and ρ_ss_(**u**) = 0 if **u** ∈ B. The states A and B are defined by the user via indicator functions in the space of the CVs. The steady-state density can be written as
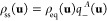
20where *q*_–_^A^(**u**) is the backward committor probability defined with the constraints *q*_A_ = 1 if **u** ∈ A and *q*_A_ = 0 if **u** ∈ B. This is verified by a direct substitution in the steady-state equation
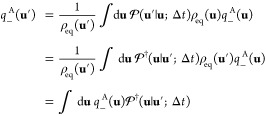
21The steady-state flux from A to B can be expressed as the net transitions across a dividing surface separating the two sides
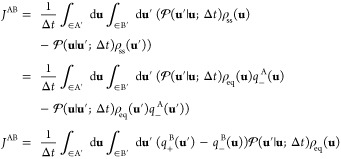
22where the integral over **u** and **u**′ are restricted to be on two different sides A′ and B′ of a separating surface between the two end states A and B, though the steady-state flux does not depend on the position of this dividing surface. Equivalently, this expression can also be obtained by combining the probability that a transition occurring from **u** to **u**′ along a reactive trajectory from A to B at equilibrium, , and the corresponding probability for the opposite transition from **u**′ to **u**, to express the net steady-state flux.

### Collective Variables and Dimensionality Reduction

Following Maragliano et al.,^6^ we introduce an approximation to the exact committor function in phase space **u** to achieve a dimensionality reduction to the subspace of the CVs **z**. We use the asantz *q̅*(**z**′) ≈ *q*_+_^B^(**u**′) and *q̅*(**z**) ≈ *q*_–_^B^(**u**) in eq 22 to express the steady-state flux *J*^AB^ and then integrate out the orthogonal degrees of freedom
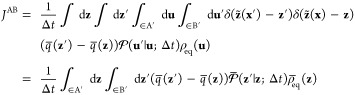
23where the effective reduced propagator is defined as
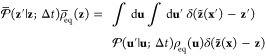
24The effective propagator obeys the detailed balance relation
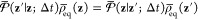
25as shown by integration of eq 13. Because the steady-state flux *J*^AB^ in eq 23 does not depend on the position of the dividing surface defining the A′ and B′ regions, it is convenient to choose a dividing surface corresponding to an isocommittor surface of *q̅*(**z**) at some arbitrary value q*. The flux from A to B can then be written as transitions from the point **z** with committor *q̅*(**z**) < q*, to the point **z**′ with committor *q̅*(**z**′) > q*.
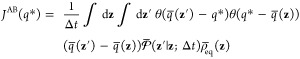
26However, one can also write
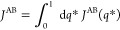
27because the steady-state flux *J*^AB^(q*) does not actually depend on the specific value of q*, which can be demonstrated by showing that there is no accumulation of probability in the region between the states A and B.^41^ Performing the integration in the expression above affects only the term
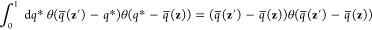
28yield the quadratic expression
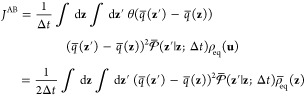
29(the factor of 2 is needed when the restriction *q̅*(**z**′) > *q̅*(**z**) is removed). An integration of the step functions analogous to eq 28 was previously used by Krivov in a different situation.^42^ A similar steady-state flux expression, quadratic in the committor difference, has also appeared in the context of discrete-state Markov models.^43^ A transformation of the steady-state flux in the phase space eq 22 through steps equivalent to eqs 26–(29) could not be found by this author. eq 29 can be used as a variational principle. The minimization of *J*^AB^ with respect to the trial function *q̅*(**z**) subject to the constraint *q̅*(**z**) = 0 when **z** ∈ A and *q̅*(**z**) = 1 when **z** ∈ B yields
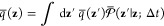
30The relation to the mean force string method via the committor defined by eqs 4–(7) can be established by assuming that the time step Δ*t* is extremely short and noting that *q̅*(**z**′) = *q̅*(**z**) + **∇***q̅*(**z**)·(**z**′ – **z**) + ..., with (**z**′ – **z**) = **z̃**(**x** + **v** Δ*t*) – **z̃**(**x**). Following through with eq 29 yields
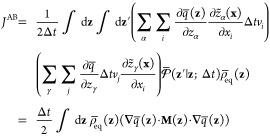
31The components of the matrix **M**(**z**), previously introduced in eq 6, are recovered from the conditional average
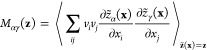
32with ⟨*v*_*i*_*v*_*j*_⟩_**z**_ = δ_*ij*_*k*_B_*T*/*m*_*i*_. The minimization of eq 31 with respect to all possible functions *q̅*(**z**) recovers eq 7, which leads to the mean force string method eq 8 of Maragliano et al.^6^ The equivalence is based on the requirement that the time step Δ*t* is extremely short.

The consequence of the dimensionality reduction to the subspace of the CVs is perhaps revealed most transparently with the following observations. The function *q̅*(**z**) is determined by the effective propagator  through eq 30. The effective propagator , according to eq 24, corresponds to a Boltzmann-weighted average of the microscopic propagator  for a single step Δ*t* with respect to the initial conditions **u** constrained by **z** and a simple sum over all states constrained **z**′. Recalling eq 7 from the analysis of the conditions for optimal dimensionality reduction of multistate Markov state models by Hummer and Szabo,^44^eq 24 is plainly recognized as a short-time local equilibrium approximation to a dimensionality reduction of the transition matrix between microstates to an effective transition matrix between macrostates. While a number of factors may be taken into consideration in attempting an optimal dimensionality reduction of a multistate Markov model,^44,45^ it is likely that this effective propagator may not be valid at a long time within the subspace of the CVs. It is legitimate to ask if the propagation within the subspace of the CVs is Markovian in the sense that . If the effective propagator is not Markovian, then eq 30 does not yield a genuine committor that represents the total probability as a sum over all possible paths starting at **z** that reach the state B before reaching the state A. For example, the forward committor defined by eq 16 implicitly assumes that a valid sum over paths can be performed as expressed by eq 15. The Markovity of the effective dynamical propagator within the reduced subspace is a necessary condition for eq 30 to yield a meaningful committor *q̅*(**z**).

### Markovity of the Effective Propagator

Let us consider the effective propagator within the subspace of the CVs for a lag time τ.
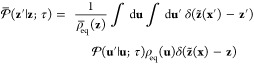
33The Markovity of the effective propagator within the subspace of the CVs implies that the Chapman-Kolmogorov equation is satisfied, . Only then, the dimensionality reduction to the subspace of CVs will yield a self-consistent representation of the dynamics of the system within this subspace (closure of the dynamical propagation). An important framework to examine this issue is to rely on a spectral decomposition of the effective dynamical propagator.^31,32^ The right-eigenvectors ψ^R^_*i*_(**z**) of the effective operator with lag time τ are defined as
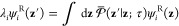
34The eigenvector ψ_1_^R^(*i*) with eigenvalue λ_1_ = 1 corresponds to an invariant state and is equal to the equilibrium vector ρ̅_eq_(**z**). There is also a set of associated orthogonal left-eigenvectors
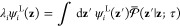
35with
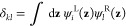
36and ψ_*i*_^L^(**z**) = ψ_*i*_^R^(**z**) ρ̅_eq_(**z**)^−1^. The first right-eigenvector is actually the equilibrium distribution ψ_1_^R^(**z**) = ρ̅_eq_(**z**).

In the context of the underlying microscopic dynamics, the validity of the effective propagator is revealed by examining the matrix element for an arbitrary time *nτ*
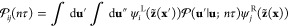
37in the context of the microscopic propagator. One can write formally the microscopic propagator as
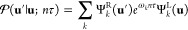
38where Ψ_*k*_^R^(**u**) and Ψ_*k*_^L^(**u**) are the genuine right- and left-eigenvector of the microscopic propagator, with Ψ_*i*_^L^(**u**) = Ψ_*i*_^R^(**u**) ρ_eq_(**u**)^−1^. As the underlying microscopic dynamics is generated from the underdamped Langevin eq 1, a spectral decomposition of the microscopic propagator  is expected to be extremely complex, reaching far beyond the scope of the present analysis. To sketch the main features of the spectral decomposition of the microscopic propagator, it is helpful to draw from a treatment of Langevin modes in macromolecules.^46^ Such an analysis indicates that the eigenvalues ω_*k*_ of the microscopic propagator can be real or complex. Complex eigenvalues come by pairs with their complex conjugate ω_k_*. The real part of these eigenvalues is negative, implying that all modes are ultimately decaying. Furthermore, a formal spectral decomposition of the Kramers-Fokker–Planck propagator indicates that its largest eigenvalues, controlling the long-time behavior, are real.^47^ One can imagine that complex eigenvalues are associated with high-frequency underdamped oscillatory motions, whereas real negative eigenvalues are associated with slow transitions and diffusive motions. There is also the existence of a time-independent stable equilibrium distribution, corresponding to a right-eigenvector with an eigenvalue of 0.
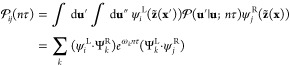
39where(ψ_*i*_^L^·Ψ_*k*_^R^) = ∫d**u**′ ψ_*i*_^L^(**z̃**(**x**′)) Ψ_*k*_^R^(**u**′), and (Ψ_*k*_^L^·ψ_*j*_^R^) = ∫d**u** Ψ_*k*_^L^(**u**) ψ_*i*_^R^(**z̃**(**x**)). With an assumption of the existence of some cutoff ω*, it is hoped that the slowest modes of the effective propagator with ∥ ω_*k*_∥ ≤ ω* will correspond accurately to the slowest modes of the microscopic propagator. It follows that (Ψ_*k*_^R^·ψ_*i*_^L^) ≈ δ_*ki*_ and (Ψ_*k*_^L^·ψ_*j*_^R^) ≈ δ_*jk*_, with the matrix elements , and the eigenvalues of the slow modes are consistent with the inherent time scales of the microscopic propagator λ_*i*_(*nτ*) = *e*^ω_*i*_ *nτ*^. In this case, the effective propagator  obeys the Chapman-Kolmogorov equation, and the propagation is Markovian within the subspace supported by this set of eigenvectors. The remaining eigenvectors correspond to faster motions with eigenvalues above the threshold, ∥ω_*k*_∥ > ω*. While those modes were determined as eigenvectors of the effective propagator with a lag time of τ, they may not accurately represent the true eigenvectors of the underlying microscopic propagator . These imperfect eigenvectors are expected to overlap with multiple fast modes of the microscopic propagator according to eq 38. As a result, a propagation within the subspace supported by those faster modes does not satisfy the Chapman-Kolmogorov equation and is not Markovian.

One solution to ensure Markovity is to project out the contribution from those modes and reconstruct the effective propagator only from the subspace of the slowest modes. This can be achieved by probing the microscopic propagator in eq 33 with a longer lag time τ, ensuring that the amplitude of the undesired contributions has sufficiently decayed away. Furthermore, as the density of fast modes is expected to be very high, a propagation for a relatively short time would rapidly cause a destructive dephasing of these contributions. In practice, one seeks to determine the smallest possible lag time that achieves Markovity for the effective propagator. The implication of this analysis is that we may have to consider a reduced propagator for a time τ longer than the microscopic time step Δ*t*. This further clarifies the origin of the apparent discrepancy between the string in CVs defined by eq 8 and the string for slow CVs defined by eq 10. The mean force string method eq 8 is derived by starting from the variational principle of eq 3 expressed in terms of the  operator associated with an infinitesimal time step in the limit of Δ*t* → 0, as shown in eq 17. As displayed through eqs 31 and (32), this makes it impossible to subsequently consider the dynamical propagation of the system over a finite lag time, which is required to reveal the conditions required to achieve Markovity in the case of slow CVs.

Once the smallest possible τ that ensures Markovity of the effective propagator has been determined, we can correctly define the forward committor for the effective dynamics within the subspace **z**
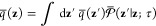
40with the constraints *q̅* = 0 when **z** ∈ A and *q̅* = 1 when **z** ∈ B. While the expression has the same form as eq 30, a critical difference is that the solution of eq 40 yields the genuine forward committor, because the effective propagator  is Markovian. This also yields the steady-state flux from A to B.
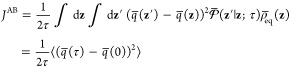
41A similar expression has been derived by Krivov and co-workers.^48,49^

It is interesting to note that the steady-state flux *J*^AB^ expressed as an equilibrium time-correlation function is related to the mean square deviation of the committor. Because the steady-state flux *J*^AB^ must be independent of the lag time τ, the form of eq 41 also indicates that the time-correlation function ⟨(*q̅*(τ) – *q̅*(0))^2^⟩ must increase linearly with τ. However, while this expression is reminiscent of the familiar expression defining the diffusion coefficient, it does not imply that the time-dependent dynamics along *q̅* is neither diffusive nor Markovian. Importantly, eq 41 can be used to construct a robust variational principle for the determination of the forward committor. Assuming a “trial” function *r*(**z**), the correct committor *q̅*(**z**) as defined by eq 40 can be found by finding the minimum of *J*^AB^ under the constraints of *r* = 0 when **z** ∈ A and *r* = 1 when **z** ∈ B. This offers a powerful route to determine optimal approximations for the committor. Eqs 33, (40), and (41) are the central results of the present dynamical propagator formulation of the transition kinetics from state A to state B.

### String Method with Swarms-of-Trajectories

In the implementation of the string method based on swarms-of-trajectories,^19^ one launches an ensemble of short trajectories from a specific position **z** corresponding to each image *m* and then calculates the mean drift of those trajectories relative to their initial starting point, ⟨Δ**z**(τ) ⟩_**z**_. The present analysis clarifies the significance of this procedure. By definition, the mean drift starting from **z** for a trajectory of length τ is
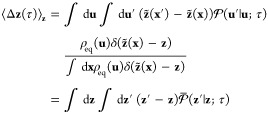
42It was shown previously that, in the limit of τ → Δ*t*, the mean drifts ⟨Δ**z**(Δ*t*) ⟩_**z**_ is equal to 1/2 Δ*t*^2^ (−**M**·**∇***W* + *k*_B_*T***∇**·**M**).^30^ It follows that the string optimized by using the mean drifts calculated with swarms-of-trajectories of length Δ*t* is equivalent to the original mean force formulation of the string method with CVs.^6^ However, as discussed above, there can be issues of non-Markovity with short trajectories in the limit of τ →Δ*t*. Intuitively, there are reasons to believe that the mean drifts from trajectories of finite time τ may better capture dissipative factors that affect the optimal pathway.^19,21^ The following analysis further clarifies this matter.

As an illustration of the consequence of a longer lag time, let us consider a case where the dynamics within the subspace of collective variables follows an overdamped Langevin equation. Presumably, the development should take the familiar form of the Smoluchowski equation. Assuming that **z**′ is close to **z**, we write *q̅*(**z**′) ≈ *q̅*(**z**) + ***∇****q̅*(**z**)·Δ**z**′(τ) with Δ**z**′(τ) = **z**′(τ) – **z**. Following through with this expansion yields
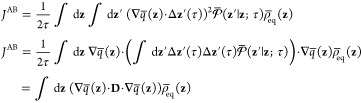
43The components of the diffusion matrix **D**(**z**) are obtained by retaining only contributions to first order in τ in the conditional average
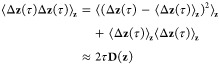
44The last term is of order τ^2^ because the mean drift is linear in τ
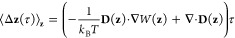
45The expansion in a small displacement also defines the forward committor for the effective dynamics within the subspace **z**, recovering the backward Smoluchowski equation previously introduced in eq 9.^41,50^ In the context of the swarms-of-trajectories, the optimized string can be determined by linking the states A and B such that the projection of the mean drifts perpendicular to the tangent of the curve vanishes.
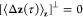
46The purpose of this analysis is to display the possible relationship of a formulation based on a dynamical propagator with lag time τ to the familiar form of the Smoluchowski diffusion equation. However, it must be emphasized that a reliance on the assumption that the CVs undergo slow diffusional dynamics is not necessary to utilize the dynamical propagator formulation embodied in eqs 33, (41), and (40).

For specific systems, it may be that the dynamics of the system within the subspace of the CVs is truly underdamped and well-characterized by an effective propagator based on a short-time approximation, while for other systems, it may be that the dynamics of the system within the subspace of the CVs is adequately represented as slow variables undergoing overdamped diffusional dynamics. The adequate dynamical regime is controlled by the underlying physics and must be ascertained for each specific system. More generally, the framework of a Markovian effective propagator with a finite lag time τ shall reflect faithfully the underlying dynamics of the system within the subspace of the CVs. In practice, the lag time τ must be chosen to represent the dynamics of the system within the subspace of the CVs as accurately as possible. The string method with swarms-of-trajectories offers a natural framework to capture the underlying dynamics with a finite lag time. A general approach to characterize the kinetics is from the perspective of a spectral analysis of the dynamical propagator .

### Variational Principle and String

A variational principle can be formulated either in terms of the left- or right-eigenvectors of the dynamical propagator  with the lag time τ.^31,32^ Assuming that we order the eigenvalues as λ_1_ > λ_2_ >···>λ_*k*_, we consider the trial left-eigenvector *v*(*i*) constructed to be orthogonal to the first (*n*–1)th left-eigenvector
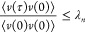
where ⟨*v*(τ)*v*(0)⟩ is an equilibrium time-correlation function. This shows that we can systematically find the *n*th eigenvector and eigenvalue, by trying to maximize the normalized time-correlation function with respect to a trial function that is orthogonal to the first (*n* – 1)th vectors. This defined the variational principle to solve the eigenvalue and eigenvector problem of the dynamical propagation operator. The formulation of the variational principle from left-eigenvector is particularly useful because the trial function is weighted by the equilibrium distribution before the propagation step, yielding directly an equilibrium time-correlation function of the trial left-eigenvector.

Let us consider the trial left-eigenvector *v*(**z**), orthogonal to the equilibrium vector and expressed as a linear combination of basis functions
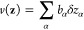
47where *δz*_α_ = (*z*_α_ – ⟨*z*_α_⟩). The orthogonality of this trial left-eigenvector to the first right-eigenvector, ∫ d**z***v*(**z**) ψ^R^_1_(**z**) = 0, is satisfied by construction. Such a linear combination of CVs is one element of the time-lagged independent component analysis (TICA),^51,52^ which was proposed to highlight kinetically relevant information in high-dimensional data. Solving for the normalized left-eigenvector corresponds to the maximization of
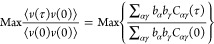
48where the equilibrium time-correlation functions are
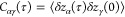
49Taking the first derivative with respect to the coefficient *b*_α_ yields
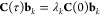
50which is a generalized eigenvalue problem yielding the *k*th left-eigenvector with the associated eigenvalue λ_*k*_.

In the context of the string method with swarms-of-trajectories,^19^ the integral over **z** weighted by the equilibrium distribution ρ̅_eq_(**z**) can be converted into a discrete sum over the images weighted by the probability *p̅*_eq_(*m*). The equilibrium time correlation function may be expressed as
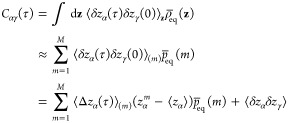
51where ⟨Δ*z*_α_(τ)⟩_(*m*)_ represents the mean drift calculated from the swarms-of-trajectories of length τ initiated at the *m*th image along the string. This suggests that the dominant dynamical behavior that determines the optimal choice of lag time is likely to arise from these terms.

### Illustrative Calculations

**Figure 2:**
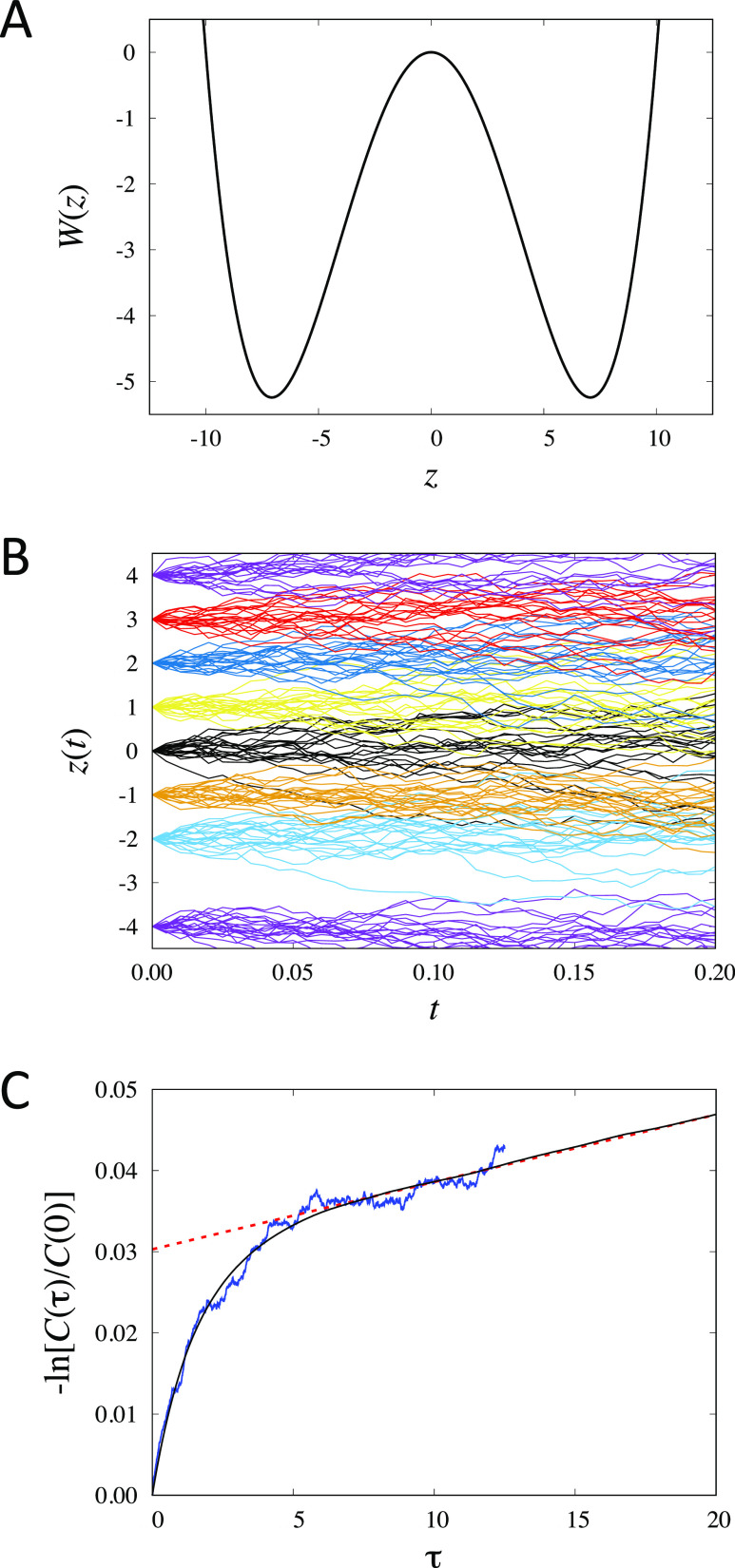
Simple one-dimensional system to illustrate the dynamics and the mean drift from swarms-of-trajectories. (A) Potential energy for the one-dimensional model. The energy surface is given by *W*(*z*) = *k*_B_*T*(0.002 0968*z*^4^ – 0.209 68*z*^2^) in units of *k*_B_*T*. (B) Swarms of 20 short Brownian dynamics trajectories initiated at specific positions along the *z* axis (diffusion coefficient is equal to 1). (C) Correlation function calculated from eq 52 (blue line) and compared with a simple average from a very long unbiased trajectory of the same system (black line). *Method:* The potential *W*(*z*) is expressed in units of *k*_B_*T*; time is expressed in reduced time units; the diffusion coefficient *D* is expressed in reduced length units squared per reduced time units. The correlation function was calculated using 201 images (one every 0.1 from from −12 to +12 in reduced units) from a swarms of 200 trajectories of 2500 steps each with a time step of 0.005. The reference correlation function was calculated from a single long unbiased trajectory of 20 million steps with a time step of 0.005.

The relationship between the mean drifts and the correlation function is illustrated with a simple one-dimensional system with two stable states separated by a free energy barrier as shown in Figure 2A. In one dimension, the trial left-eigenvector *v*(*z*) defined by eq 47 is simply *δ z* = (*z* – ⟨*z*⟩). In this simple case
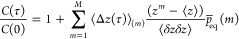
52In this development, it is assumed that the equilibrium distribution of the images *p̅*_eq_(*m*) has been determined. Generally, this can be done efficiently using biased sampling methods.^53,54^Eq 52 clarifies the significance of the mean drifts ⟨Δ*z*(τ)⟩_(*m*)_ in the string method with swarms-of-trajectories.

The behavior of the swarms is depicted in Figure 2B. With the quantity [*C*(τ)/*C*(0)] = λ_2_ to monitor the slowest eigenvalue, Markovity should mean that the implicit relaxation rate equal to −ln λ_2_/τ is independent of the lag time τ. However, the simple functional form *δz* is not a perfect trial eigenvector, and, as illustrated in Figure 2C, this condition is only met for a lag time larger than 10 reduced time units, which reflects mainly the relaxation of the two-state system. This is still much shorter than the slowest relaxation time of the double-well system, which is on the order of 1200 reduced time units. Nevertheless, this example shows that it is possible to calculate an equilibrium time-correlation function from controlled starting configurations.

The slow convergence suggests that it is important to go beyond a description that relies mainly on a linear combination of CVs to represent the dynamics along the string. In a more realistic multidimensional system, it is possible that the optimal string could be established for a shorter lag time using a richer basis set that relies on the images along the string pathway. For example, the images along the string can be used to define “one-hot” indicator functions in the subspace of the CVs, leading to the traditional Markov State (MSM) formulation.^55^ Likewise, the images along the string can be used to support a Voronoi tessellation in the context of a milestoning algorithm.^56−58^ A future application should exploit the position of the individual images as kernels to construct the basis set using nonlinear functions.

**Figure 3:**
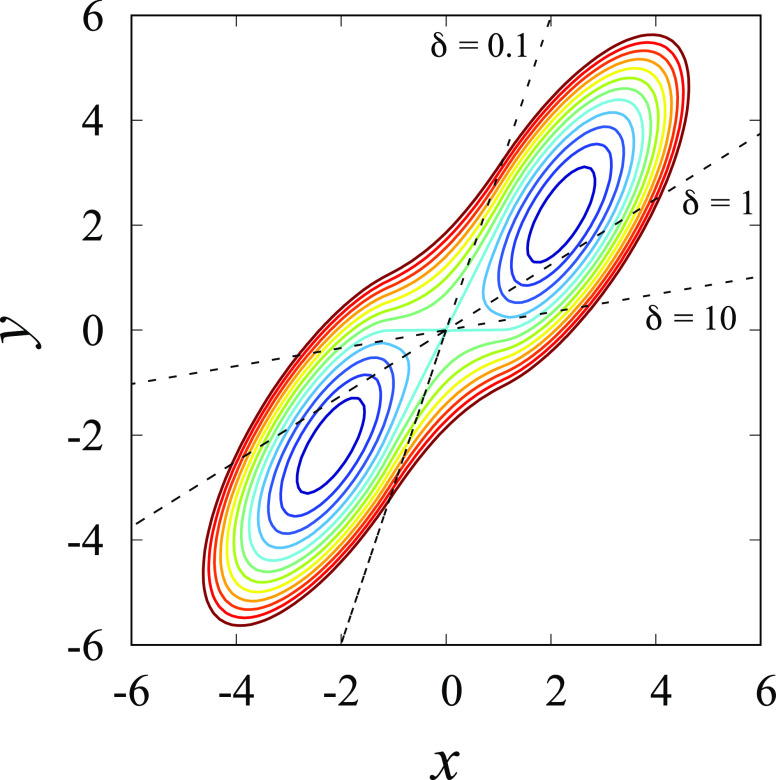
Potential *W*(*x*,*y*) of the 2D system was taken from eqs (14a) and (14b) of Berezhkovskii and A. Szabo.^59^ The energy surface is in units of *k*_B_*T*, and the levels are at −4.0, −3.0, −2.0, −1.0, 0.005, 1.0, 2.0, 3.0, 4.0, 5.0, 6.0, and 7.0. The dash lines represent the direction of the dominant reactive mode for the different conditions: θ = 32° for δ = 1, θ = 72° for δ = 0.1, and θ = 10° for δ = 10.

This exceedingly simple one-dimensional model serves to illustrate the relationship between the mean drifts and the correlation function reflecting the inherent time scales within the system. However, the directionality of the path is one critical feature cannot be captured in one dimension. This can be illustrated with the simple two-dimensional (2D) system with two stable states separated by a free energy barrier shown in Figure 3. The same model was previously used by Berezhkovskii and Szabo to examine the effect of anisotropic diffusion^59^ and also by Tiwary and Berne to illustrate the spectral gap optimization of order parameters (SGOOP) method for predicting reaction coordinates.^60^ The magnitude of the anisotropy is characterized in terms of the parameter δ = *D*_*y*_/*D*_*x*_, where *D*_*x*_ and *D*_*y*_ are the diffusion coefficients along the *x* and *y* axes, respectively. The dash lines in Figure 3 represent the direction of the dominant reactive mode for three different values of δ. The MFEP corresponds to the dominant reactive path (θ = 32°) only when the diffusion is isotropic (δ = 1).^59^ To capture the directionality of the path through the saddle point, we model the committor in the space of the CVs by the simple function
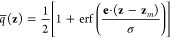
53with **z** ≡ (*x*,*y*) in two dimensions. The normal to the isocommittor surface is given by the unit vector **e**; the width of the separatrix region (*q* = 0.5) located at **z**_*m*_ is given by the parameter σ. By construction, this form starts with *q* = 0 in the reactant region A and reaches *q* = 1 in the product region B. eq 53 can be derived from a quadratic barrier (see Appendix). Since we are only interested in the direction of the path here, we used the knowledge that the top of the barrier is located at **z**_*m*_ = (0,0) for the sake of simplicity. In two dimensions, the unit vector **e** can be characterized via the angle θ with the *x*-axis.^59,60^ Similar functional forms were previously used by Peters and Trout to optimize reaction coordinates via likelihood maximization.^61,62^

**Figure 4:**
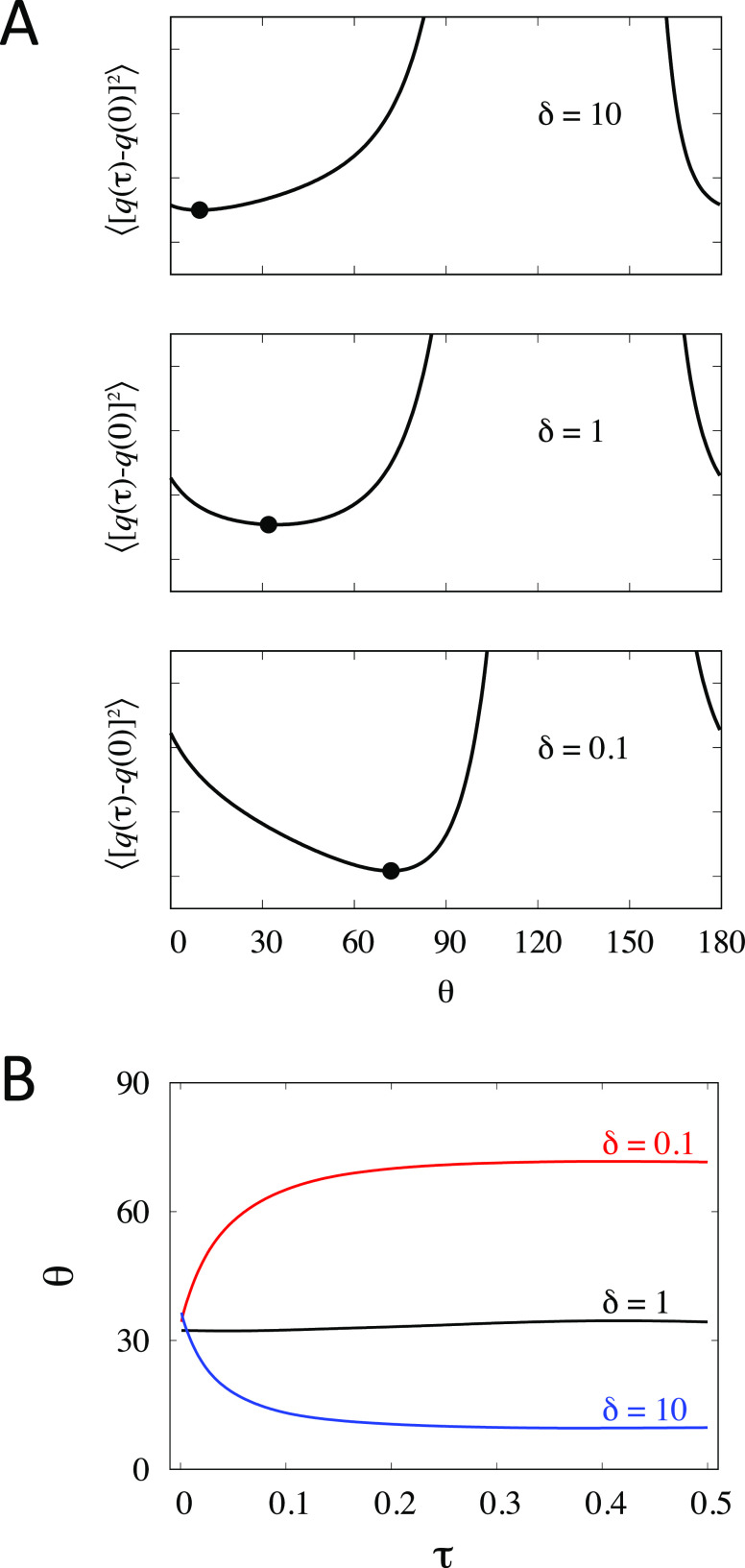
Results from using the reactive flux correlation function from eq 41 with the committor based on eq 53. (A) Dependence of the time-correlation reactive flux function ⟨[*q*(τ) – *q*(0)]^2^⟩ on the angle θ for the direction of the path. The maximum of the curve occurring around θ = 130° (orthogonal to the axis linking the two wells) is not shown for clarity. The time-correlation function is shown for a fixed lag time τ = 0.5. The black circle indicates the position of the minimum in the curves: θ = 32° for δ = 1, θ = 72° for δ = 0.1, and θ = 10° for δ = 10. (B) Dependence of the time-correlation reactive flux function ⟨[*q*(τ) – *q*(0)]^2^⟩ on the lag time τ (the optimal angle θ is used for each case). The width parameter σ is equal to 1.0 in all cases. *Method:* The time-correlation functions were calculated from a Langevin dynamics trajectory of 10 million steps generated via eq 1, with γ_*x*_ = *D*_*x*_/*k*_B_*T* and γ_*y*_ = *D*_*y*_/*k*_B_*T*, for *D*_*x*_ and *D*_*y*_ taking values of 1.0 and 0.1 corresponding to an anisotropy δ = *D*_*y*_/*D*_*x*_ of 0.1, 1.0, and 10.0. The mass was chosen to yield relaxation times γ/*m* of 0.08 and 0.008 reduced time unit, for diffusion coefficients of 1.0 and 0.1, respectively.

On the basis of the model committor based on eq 53, we determine the direction of the optimal path by seeking to minimize the time-correlation function ⟨[*q*(τ) – *q*(0)]^2^⟩ as a function of the angle θ. Following previously work,^59,60^ we compare three cases with δ = 1 (*D*_*x*_ = 1.0 and *D*_*y*_ = 1.0), δ = 10 (*D*_*x*_ = 0.1 and *D*_*y*_ = 1.0), and δ = 0.1 (*D*_*x*_ = 1.0 and *D*_*y*_ = 0.1). Upon examination, the optimal value of the width parameter σ was found to vary only slightly between 0.8 and 1.0; it was kept equal to 1.0 in all cases for the sake of simplicity. The results are shown in Figure 4. In all cases, the minimum of ⟨[*q*(τ) – *q*(0)]^2^⟩ yields the correct direction of the reactive paths previously identified (Figure 4A).^59,60^ It is noteworthy that this formal treatment based on the minimization of the time-correlation function ⟨[*q*(τ) – *q*(0)]^2^⟩ does not require any assumptions regarding the Markovity of the dynamics along the committor itself. This contrasts with other methods assuming that the dynamics along the putative optimal path is Markovian.^60−62^ While the present formulation requires that the effective propagator  within the subspace of the CVs be Markovian (see above), this is not generally true of the dynamics projected long the one-dimensional committor coordinate.^41^

Of particular interest is the dependence of the reactive path direction as a function of the lag time τ. As shown in Figure 4B, clearly all three reactive paths appears to converge to the isotropic case (δ = 1 and θ = 32°) when the lag time is extremely short. The small discrepancy between the three cases at τ ≈ 0 is due to the magnitude of the dissipation;it vanishes if *D*_*x*_ and *D*_*y*_ are increased while keeping δ constant. In the isotropic case, the reactive path captured by the short-time propagation corresponds to the MFEP. This path is equivalent to the mean-force string method as prescribed by eqs 5, (6), (8), (31), and (32).^6,30^ The short-time propagator is effectively “blind” to the true dissipative nature of the microscopic dynamics of the CVs (Figure 4B). The optimal directionality of the path only emerges as the lag time is increased to 0.5 reduced time units. As prescribed by the minimization of the time-correlation function ⟨[*q*(τ) – *q*(0)]^2^⟩, the correct path follows the true slow reactive mode, which is dominated by the smallest diffusion coefficient; if *D*_*x*_ < *D*_*y*_ the path follows the *x*-axis (θ is close to 0°), and if *D*_*x*_ > *D*_*y*_ the path follows the *y*-axis (θ is close to 90°).

This analysis suggests a potentially practical “staging” strategy for characterizing the direction of the reactive paths in a multidimensional systems with a single dominant barrier. Operationally, the path is prescribed by minimizing the equilibrium time-correlation function ⟨[*q*(τ) – *q*(0)]^2^⟩. In effect, minimizing this quantity is associated with discovering the region in the subspace of the CVs where the fluctuations over *q* over a lag time τ are as infrequent as possible. Motivated by this perspective, we consider the harmonic restraining potential *u*_*m*_(**z**) = 1/2*k*(**z** – **z**_*m*_)^2^ and rewrite the time-correlation function for the reactive flux as
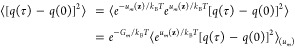
54where ⟨···⟩_(*u*_*m*_)_ indicates a conditional average initially weighted by the restraining potential *u*_*m*_, and *G*_*m*_ = −*k*_B_*T* ln⟨exp[−*u*_*m*_(**z**)/*k*_B_*T*]⟩ is the free energy for introducing the restraining potential *u*_*m*_.^63^ For example, similar restraining potentials are applied on the *M* images in the string method with swarms-of-trajectories.^19^ In a staging strategy to minimize the reactive flux time-correlation function, one should first seek to locate the position **z**_*m*_ that yields the largest *G*_*m*_ and then determine the optimal direction of the reactive path from the conditional time-correlation function ⟨*e*^*u*_*m*_(**z**(0))/*k*_B_*T*^[*q*(τ) – *q*(0)]^2^⟩_(*u*_*m*_)_ for a functional model of *q* such as that given by eq 53. Even though the biasing factor exp[*u*_*m*_(**z**)/*k*_B_*T*] in eq 54 increases the weight of the configuration at large distances from the top of the barrier, the conditional average is expected to converge locally because the quantity [*q*(τ) – *q*(0)]^2^, which goes as **∇***q*·**D**·**∇***q* in the diffusive limit according to eq 43, is nonzero only in the region where the committor varies abruptly. Correspondingly, the contribution from regions away from the barrier top where the committor is nearly constant are expected to be negligible. A framework akin to Chandler’s reactive flux formalism is essentially recovered in the limit where the model committor varies abruptly as a Heaviside step-function.^64^

Simple tests with the 2D potential surface of Figure 3 indicate that the staging strategy can work. Using the restraining potential *u*_*m*_ = 3*k*_B_*T*(*x*^2^ + *y*^2^) to prepare initial conditions near the barrier top, followed by a swarms of 10 000 unbiased short trajectories of 0.5 time units (500 steps) show that the staging method can yield correct results for the direction of the reactive paths, with angles of θ = 32° for δ = 1, θ = 68° for δ = 0.1, and θ = 18° for δ = 10. While these results on a simple model are encouraging, more work is needed to further develop the staging strategy into a fully practical and reliable method.

## Conclusion

We have formulated the problem of the kinetics of a dynamical system comprising two metastable states A and B in terms of a finite-time propagator in phase space (position and velocity) adapted to the underdamped Langevin equation. The present development expands on a previous formulation based on propagators for position-based Markov models.^31−34^ Starting from the full phase space representation with positions and velocities, the central ansatz assumes that the committor depends only on the variables of the selected collective variables. The dimensionality reduction to the subspace of CVs yields familiar expressions for the propagator, committor, and steady-state flux, as embodied in eqs 33, (41), and (40), respectively. These are the central results of the development. Importantly, eq 40 is a quadratic expression for the steady-state flux *J*^AB^ between the metastable states A and B that can serve as a robust variational principle to determine the optimal committor. These expressions can be exploited in the context of the string method, in which the dominant pathway between A and B is represented by a curve in the space of the CVs, to calculate dynamical correlation.

The analysis based on the effective dynamical propagator in the reduced space of the CVs, , clarifies the theoretical foundation of the string method with swarms-of-trajectories to determine optimal transition pathways.^19^ Most importantly, the analysis revealed the fundamental significance of the lag time to calculate the mean drift during short unbiased trajectories.^19,21^ The lag time τ must be chosen so to satisfy the conditions of Markovity the effective propagator  within the subspace of the CVs. In practice, τ should be as short as possible but sufficiently large to ensure that  is Markovian. The string method with swarms-of-trajectories offers a natural framework to capture the underlying dynamics within the subspace of the CVs. If this dynamics experiences very little dissipative coupling and is near-inertial, then it is possible that Markovity is attained with a very short lag time (short swarms-of-trajectories). In this case, the mean drifts from the swarms-of-trajectories is essentially equivalent to the original mean force string method.^6,30^ However, if the effective dynamics within the subspace of the CVs experiences complex dissipative effects, then a longer lag time is needed to guaranty the Markovity of the effective propagator—a necessary condition to determine the genuine forward committor . Without Markovity, the solution *q̅*(**z**) of eq 30 based on the effective short-time propagator  is not the forward committor in the subspace of the CVs. In both cases, the mean drift from the swarms-of-trajectories correctly captures the effective dynamical behavior of the system within the subspace of the CVs, supporting the construction of a meaningful transition pathway.^19,21^ The formulation of the propagator with a finite lag time makes it possible to determine the necessary conditions for Markovity and the progression of the committor along the path. Furthermore, it circumvents the need to rely on the rank ordering of the different images along the string to define a one-dimensional effective reaction coordinate.^65,66^ Interestingly, alternate methods built upon an optimized pathway, such as Milestoning^56,67^ or MSM,^55^ also offer the possibility to directly examine the natural unbiased dynamics within the subspace of the CVs. Furthermore, the effective propagator  with finite lag time τ is amenable to an eigenvalue-eigenvector spectral analysis as elaborated previously in the context of position-based Markov models.^31−34^ The time-correlation functions calculated by swarms-of-trajectories along the string constitutes a natural extension of these developments. The present analysis is also related to a recent dynamical Galerkin approximation (DGA) formulated to predict the long-time scale behavior from short-trajectory.^68,69^

The present formulation strengthens the theoretical framework to determine the optimal pathway between the states A and B, that is, the proverbial “reaction coordinate”, and to characterize the long-time kinetics of the system. Identifying the most relevant subspace of CVs and then determining the most representative reaction coordinates in this subspace remains the greatest challenge. In this endeavor, other related ideas may be brought to bear, including a dynamical self-consistency,^70^ memory reduction,^71,72^ multidimensional spectral gap optimization of order parameters (SGOOP),^73,74^ and maximally predictive one-dimensional projection.^33^ Of particular interest are the ideas of Krivov, who proposed a nonparametric variational optimization of reaction coordinates.^75^ Future work will further explore these ideas.

## Appendix: Committor and Quadratic Barrier

Following Berezhkovskii and Szabo,^41^ we assume that the top of the barrier at **z** = 0 is a multidimensional quadratic form, *βW* = **z**^*t*^**Vz**/2 and that the committor can be written as *q*(**z**) ≈ *q*(**e**·**z**) in the neighborhood of the saddle point (we drop the overline on *q* to simplify the notation). At this point, **e** is an unknown unit vector that has yet to be determined. The gradient Laplacians of *q* are **∇***q*(**z**) = **e***q*′(**e**·**z**) and **∇**^2^*q*(**z**) = *q*″(**e**·**z**), respectively. Starting from the backward Kolmogorov eq 9,^41,50^ in the case of a constant (independent of position) anisotropic diffusion matrix **D**, we have

where *s* = **z**·**e**. To close this equation with respect to *s*, we require that the vector **e** be chosen to satisfy the condition

(λ > 0). Using **z**^*t*^**e** = **e**·**z** = *s*, we can write

Letting **e**·**D**·**e** = *D*, we write

leading to

with σ^2^ = 2*D*/λ. This functional form is similar to eq 3.8 from Berezhkovskii and Szabo that was obtained following a different route.^41^
